# Supplementary material for: An Efficient Synthesis of Acenaphtho[1,2-b]indole Derivatives via Domino Reaction
Source: Molecules. 2018 Nov 21;23(11):3045. doi: 10.3390/molecules23113045 (PMC6278500; doi:10.3390/molecules23113045)
Supplement: Supplementary file 1 [file molecules-23-03045-s001.pdf]

Supplementary Material

An Efficient Synthesis of Acenaphtho[1,2-*b*]indole Derivatives via Domino Reactions

Guo-Ning Zhang [1,†], Xia Yuan [2,†], Weiping Niu [1], Mei Zhu [1], Juxian Wang [1,*] and Yucheng Wang [1,*]

1 Institute of Medicinal Biotechnology, Chinese Academy of Medical Science and Peking Union Medical College, Beijing 100050, China; raisunny2006@163.com (G.-N.Z.); niuweip@126.com (W.N.); mzhu87@163.com (M.Z.)
2 State Key Laboratory of Natural and Biomimetic Drugs, School of Pharmaceutical Sciences, Peking University, Beijing, 100191, PR China; yuanxia@bjmu.edu.cn
† Both authors contributed equally to this work.
* Correspondence: imbjxwang@163.com (J.W.); wyc9999@126.com (Y.W.); Tel.: +86-10-63131053 (J.W.); +86-10-6316-5263 (Y.W.)

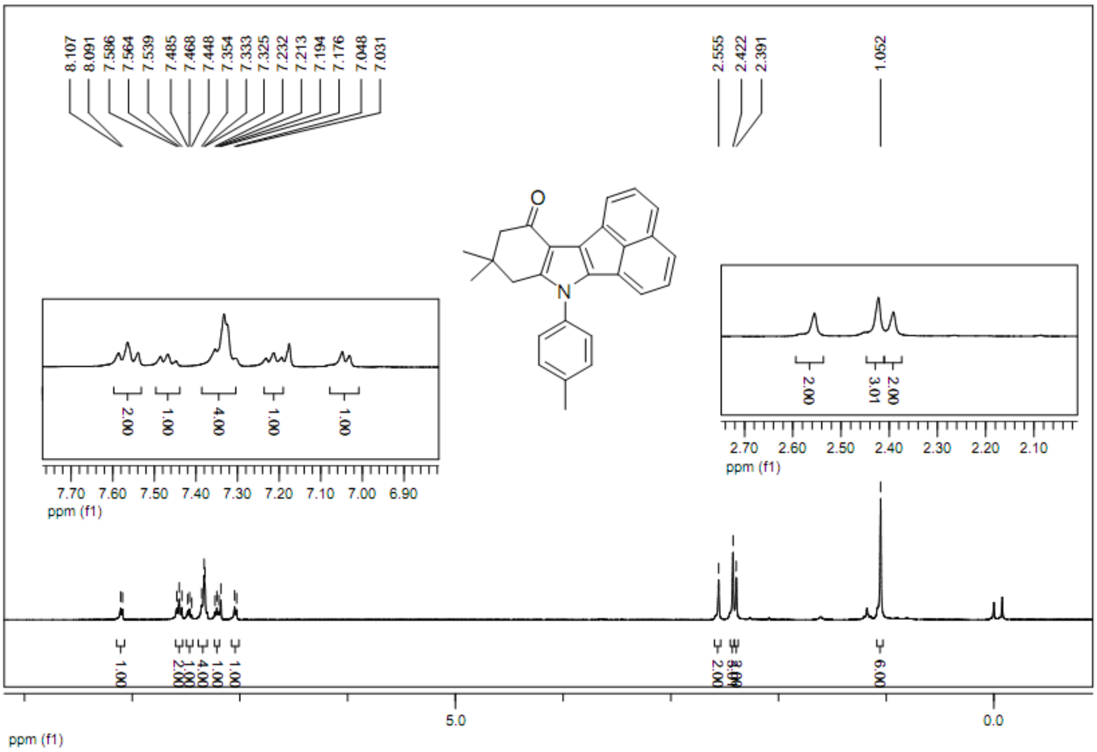

Figure S1. ^{1}H-NMR spectrum of compound **3a**

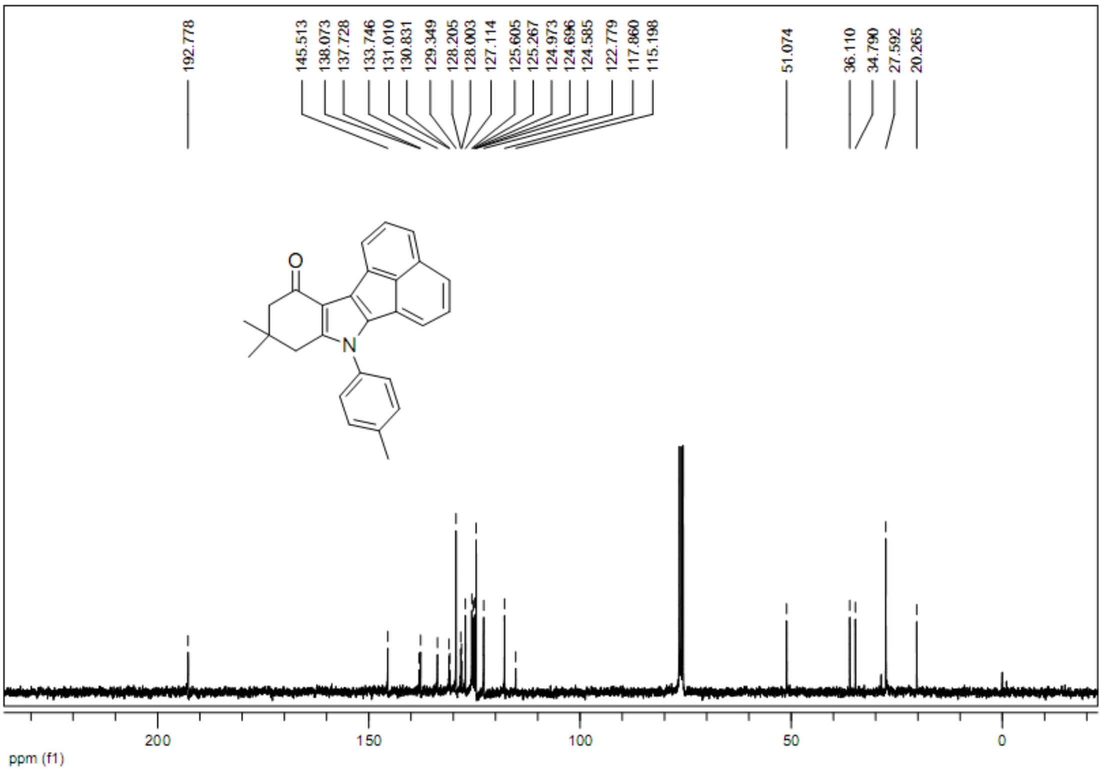

Figure S2. ^{13}C-NMR spectrum of compound **3a**

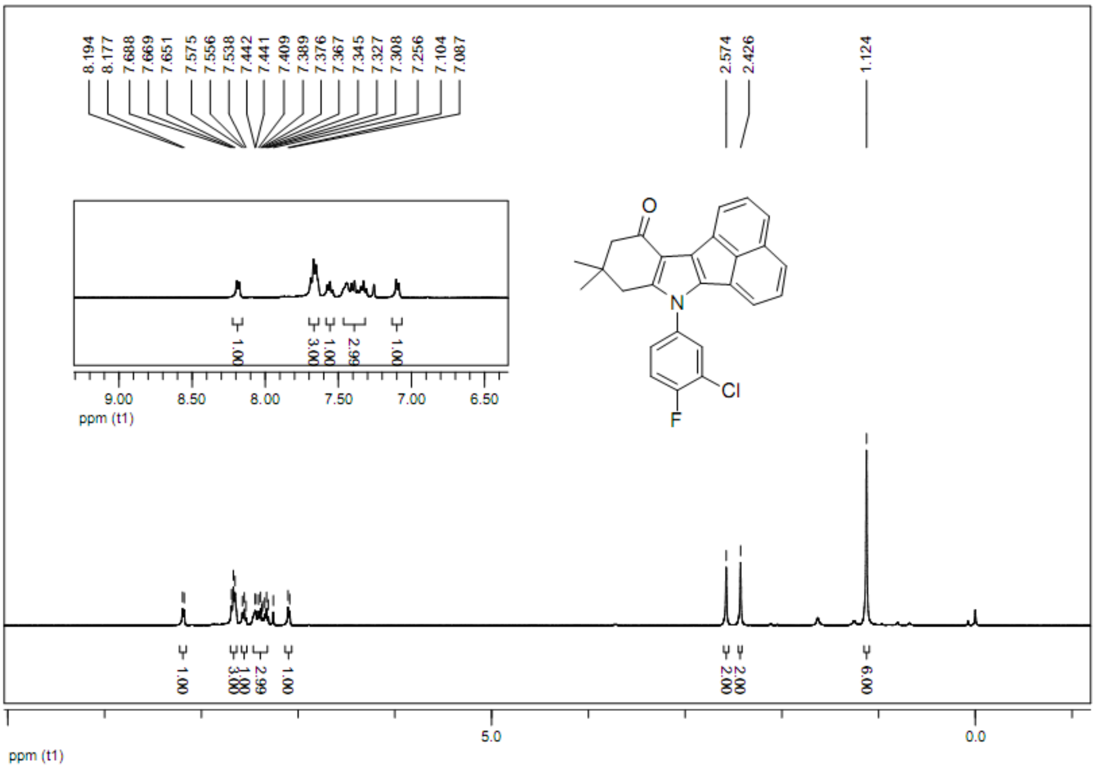

Figure S3. ^{1}H-NMR spectrum of compound **3b**

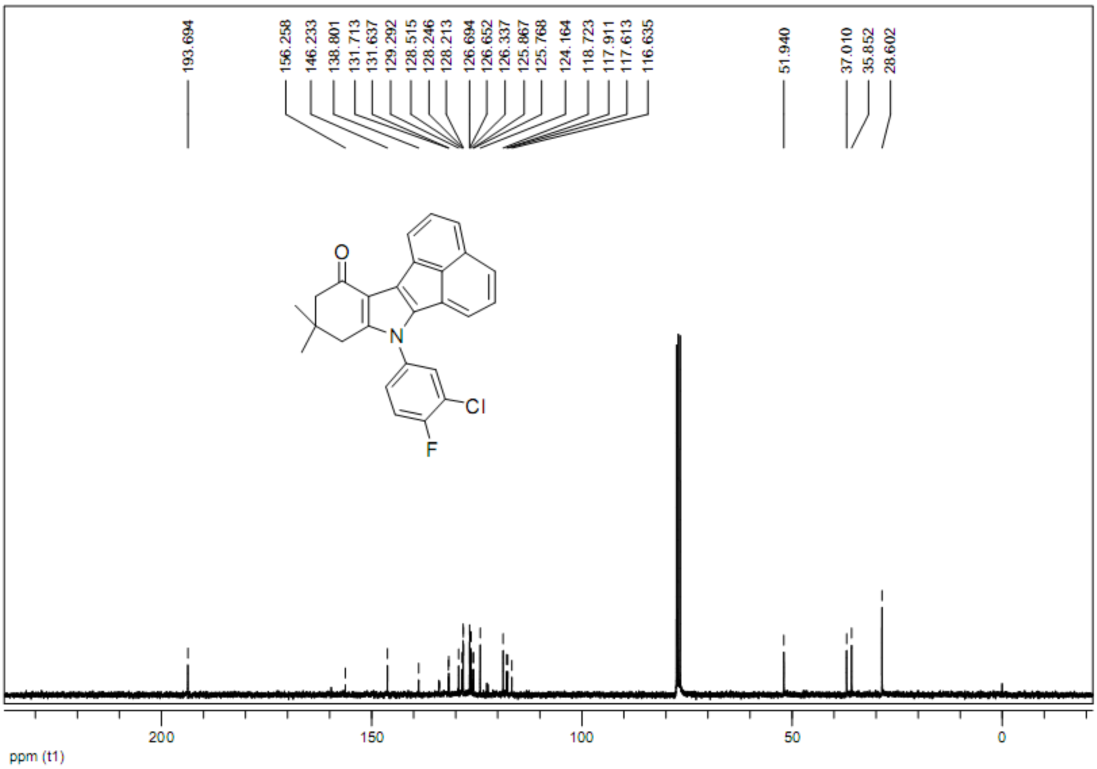

Figure S4. ^{13}C-NMR spectrum of compound **3b**

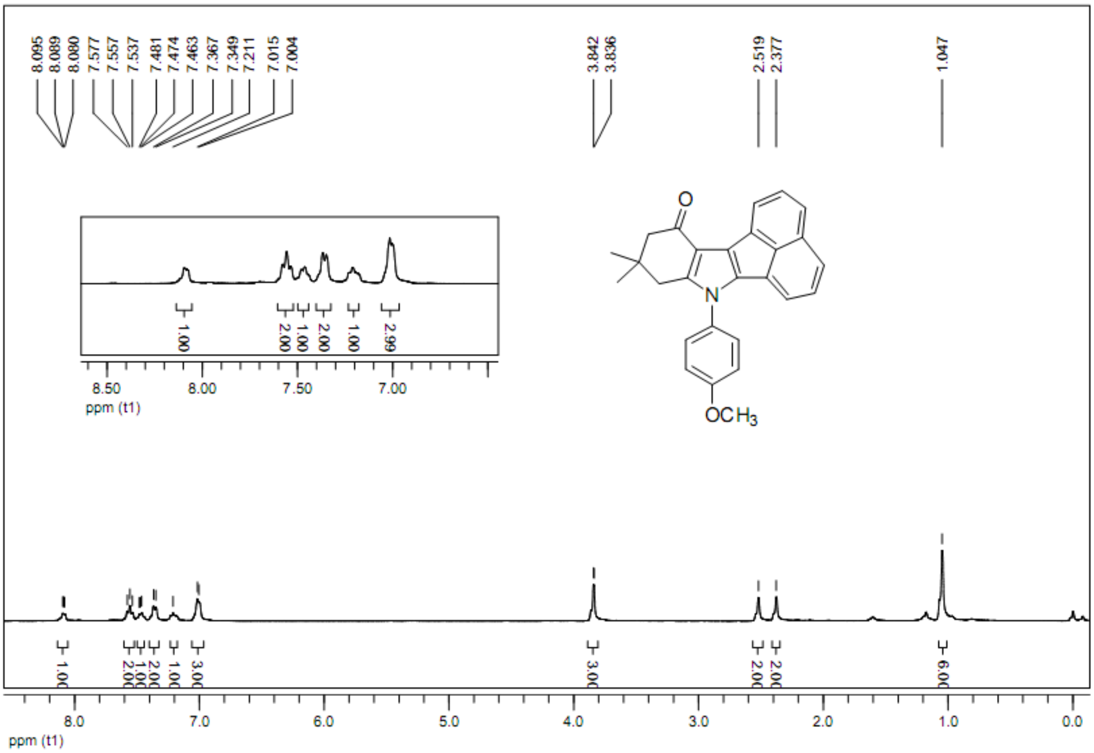

Figure S5. ^{1}H-NMR spectrum of compound **3c**

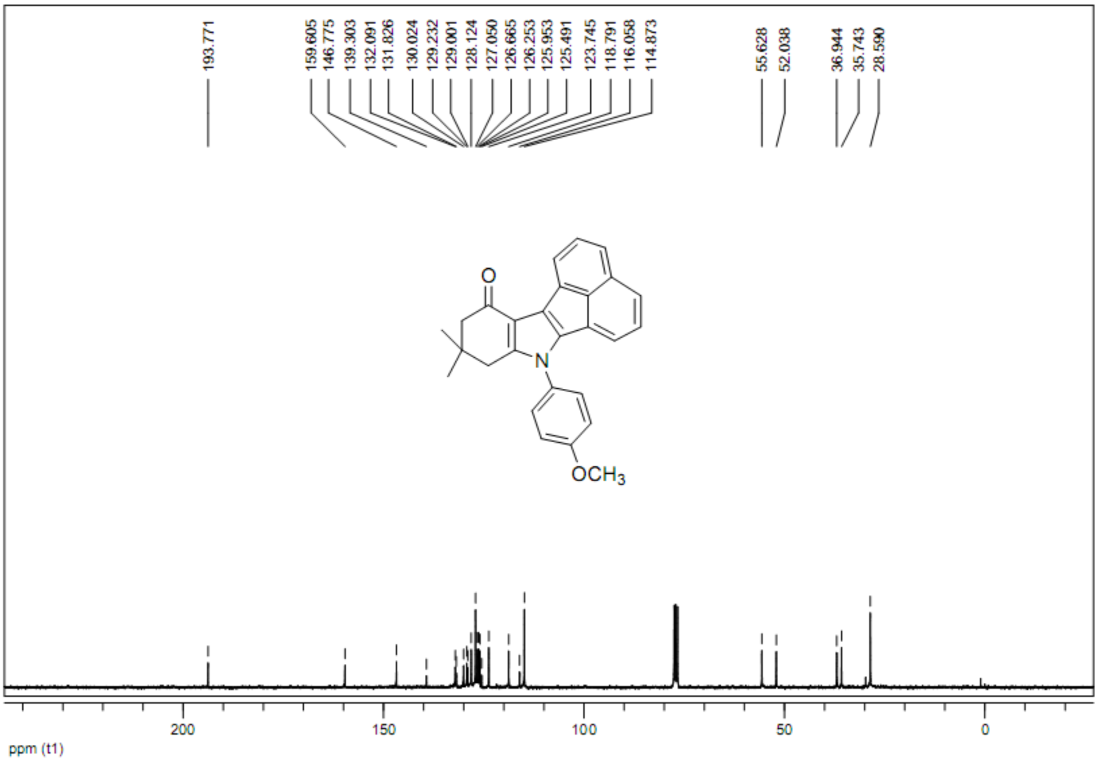

Figure S6. ^{13}C-NMR spectrum of compound **3c**

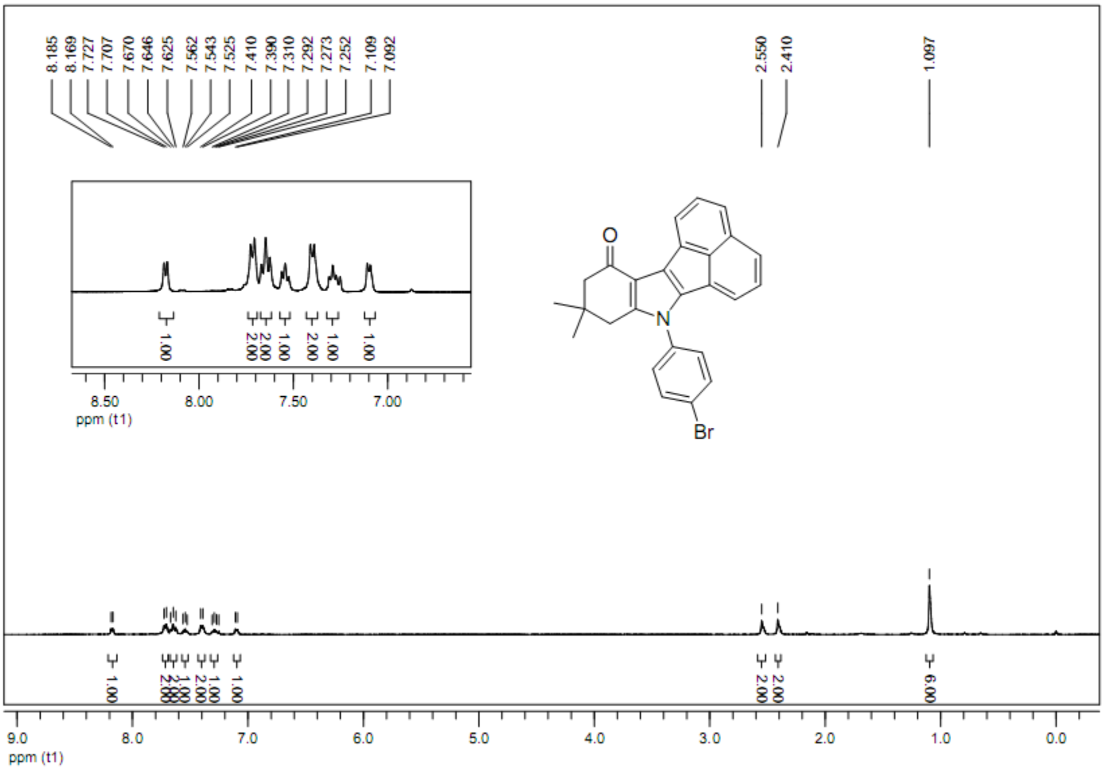

Figure S7. [13]H-NMR spectrum of compound **3d**

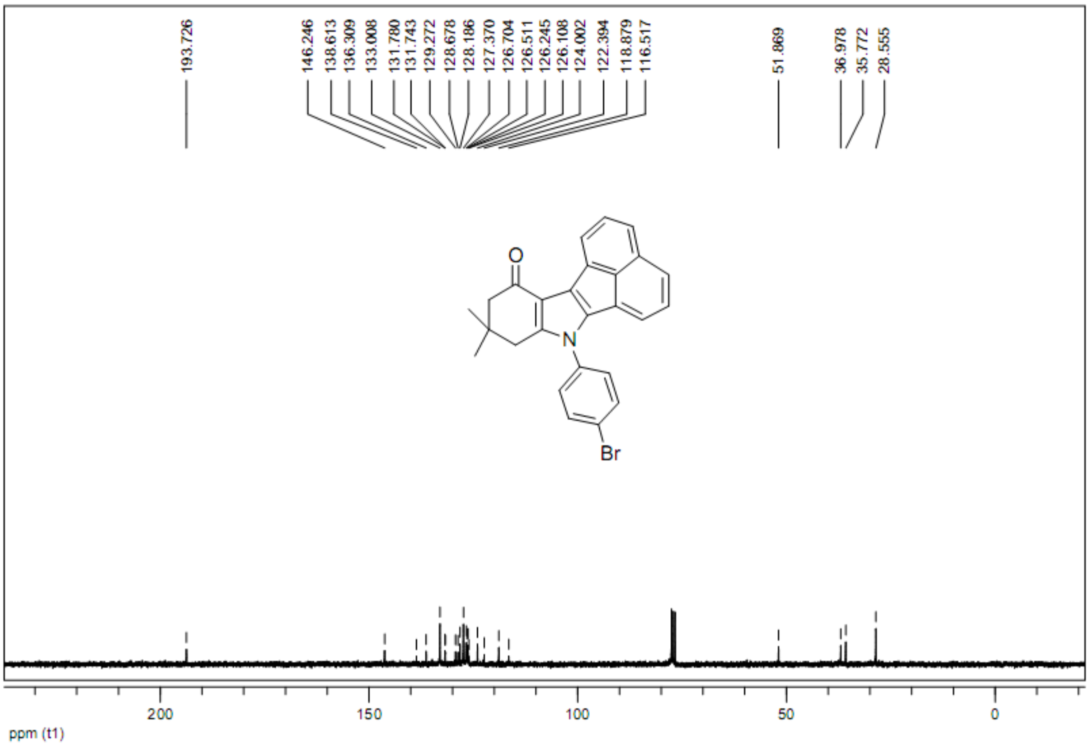

Figure S8. ^{13}C-NMR spectrum of compound **3d**

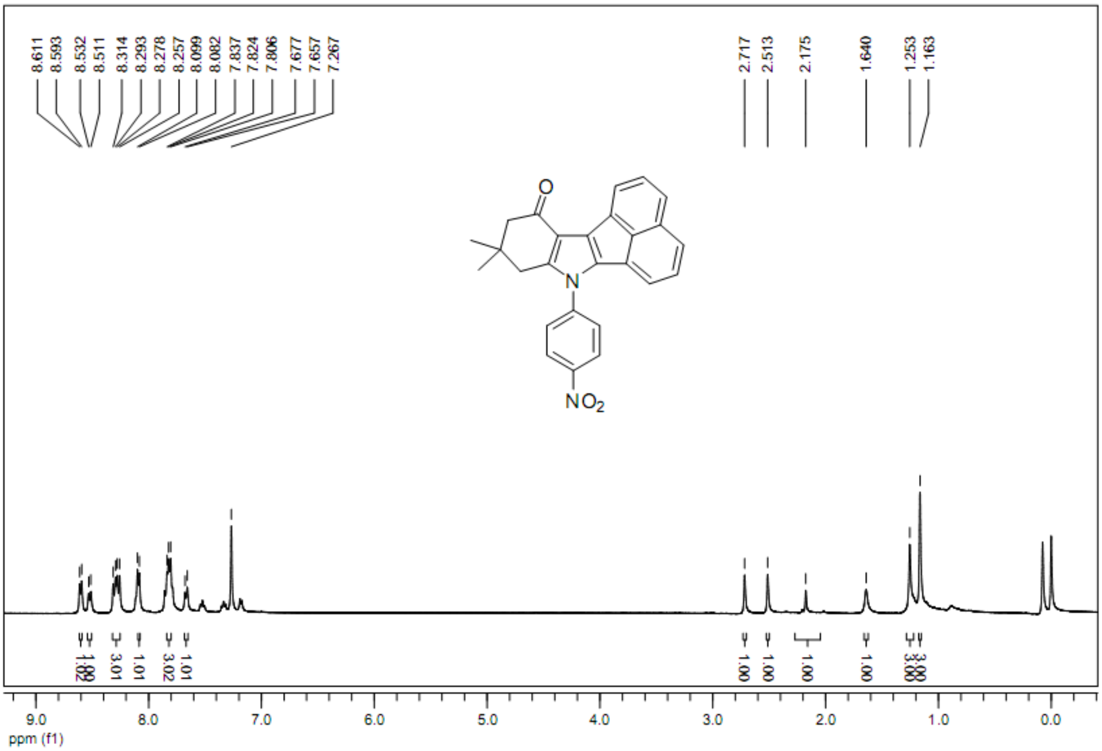

Figure S9. ^{13}H-NMR spectrum of compound **3e**

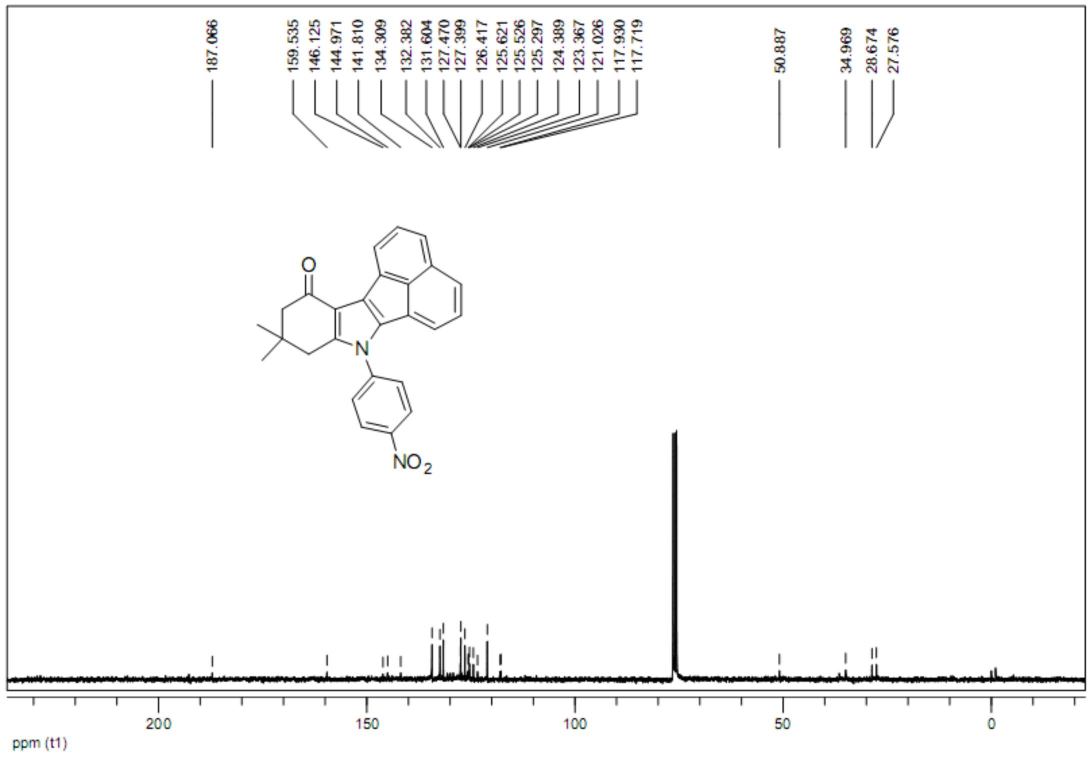

Figure S10. ^{13}C-NMR spectrum of compound **3e**

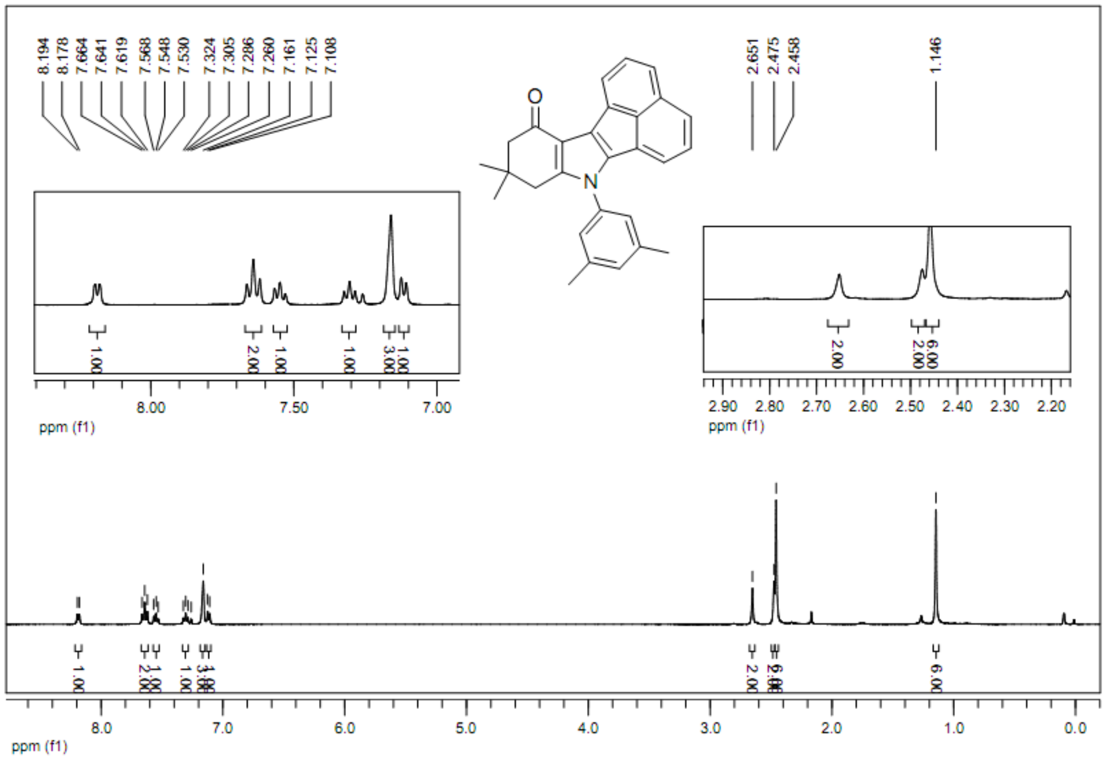

Figure S11. ^{1}H-NMR spectrum of compound **3f**

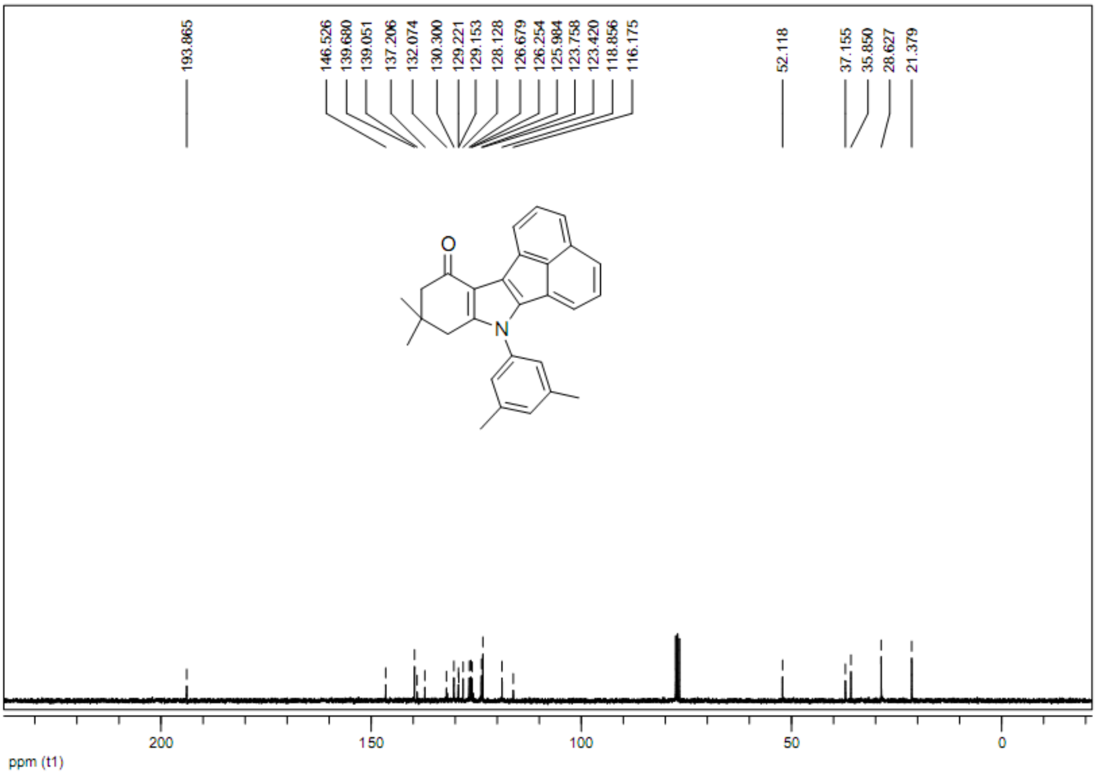

Figure S12. ^{13}C-NMR spectrum of compound **3f**

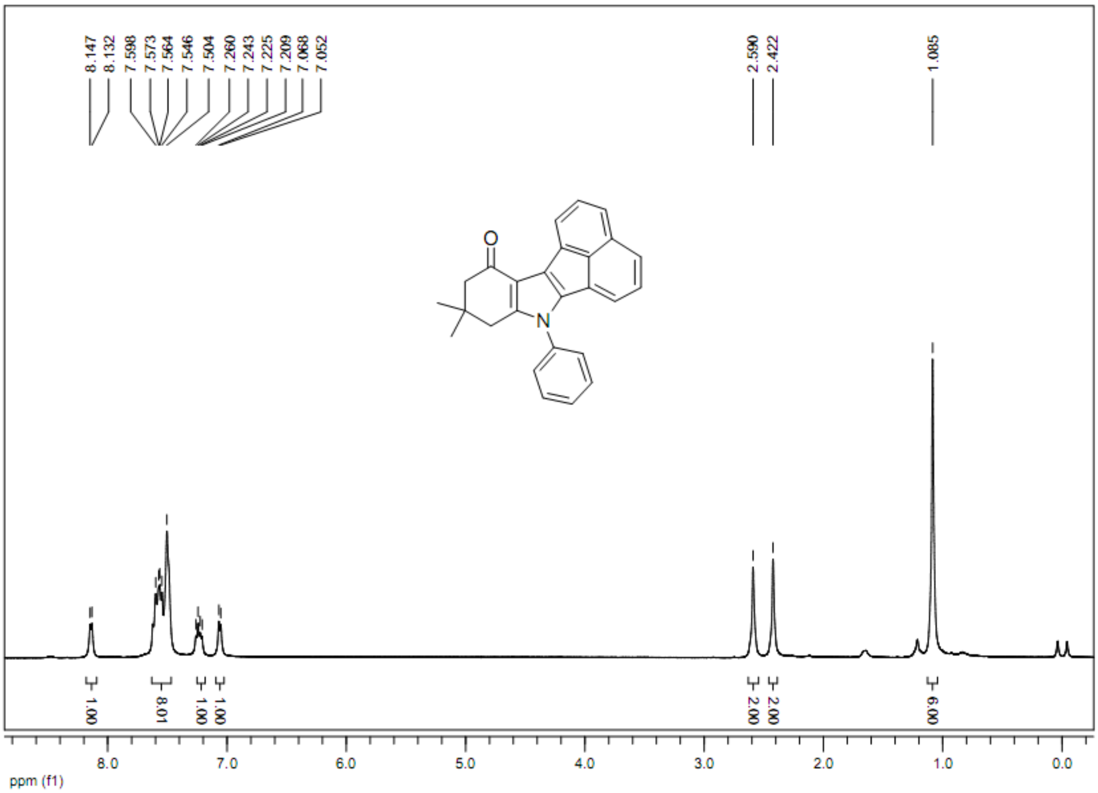

Figure S13. ^{1}H-NMR spectrum of compound **3g**

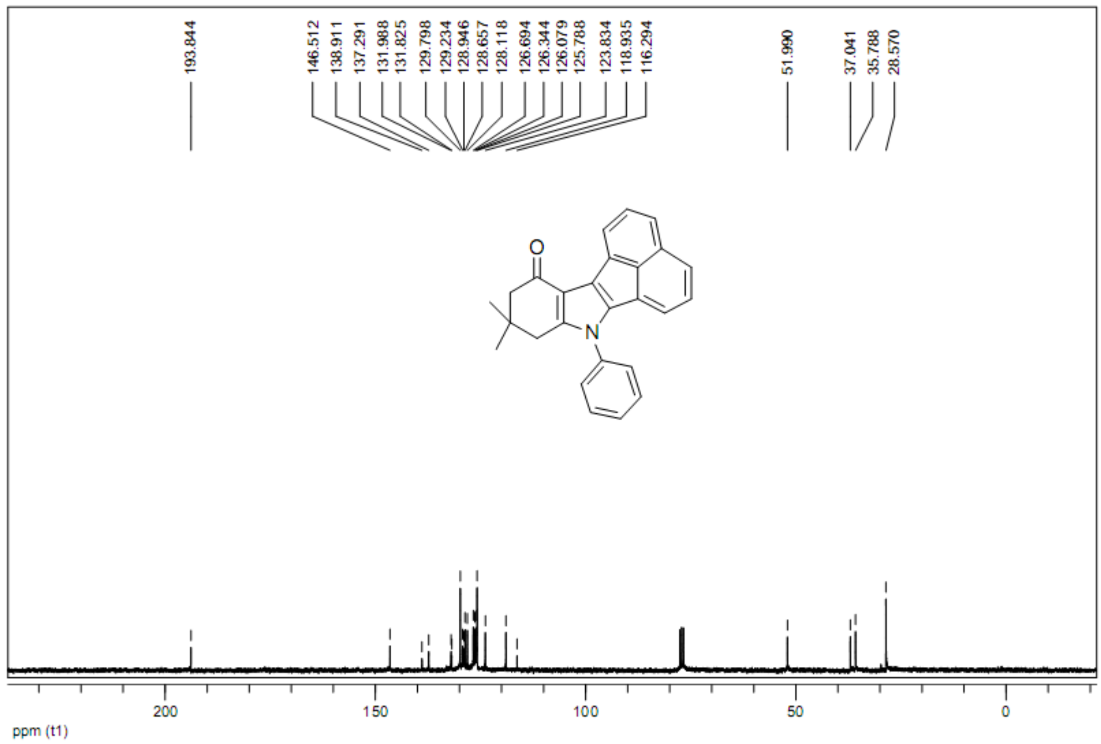

Figure S14. ^{13}C-NMR spectrum of compound **3g**

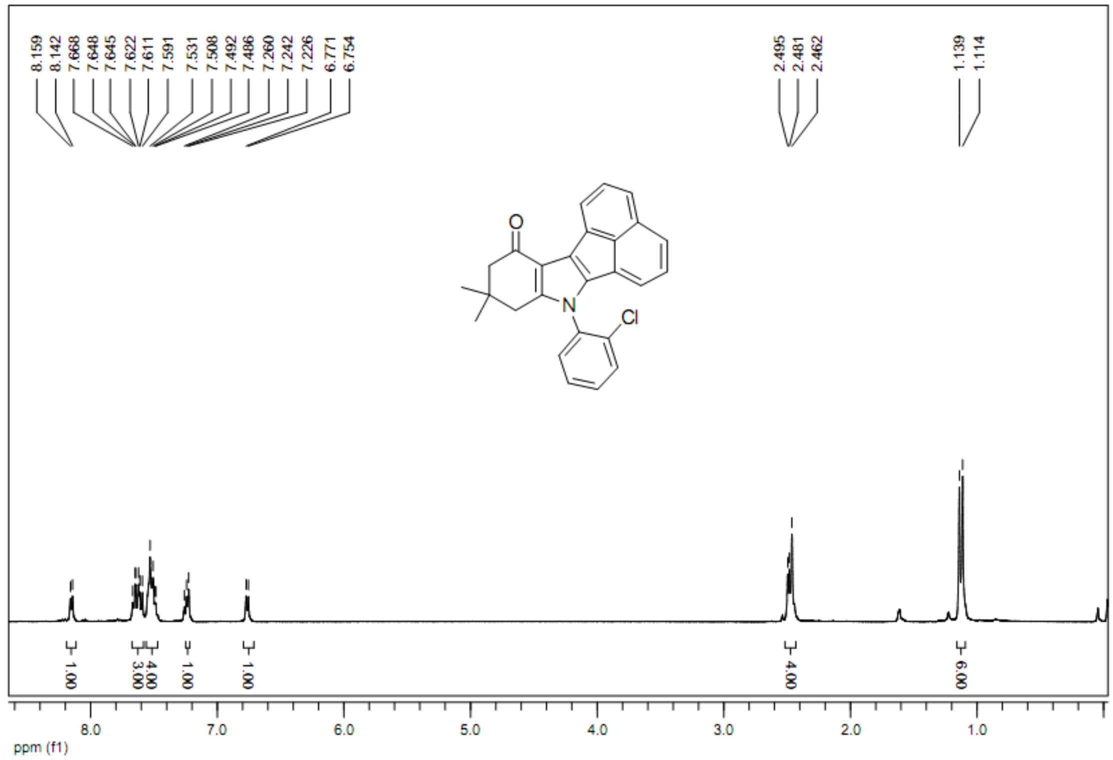

Figure S15. ^{1}H-NMR spectrum of compound **3h**

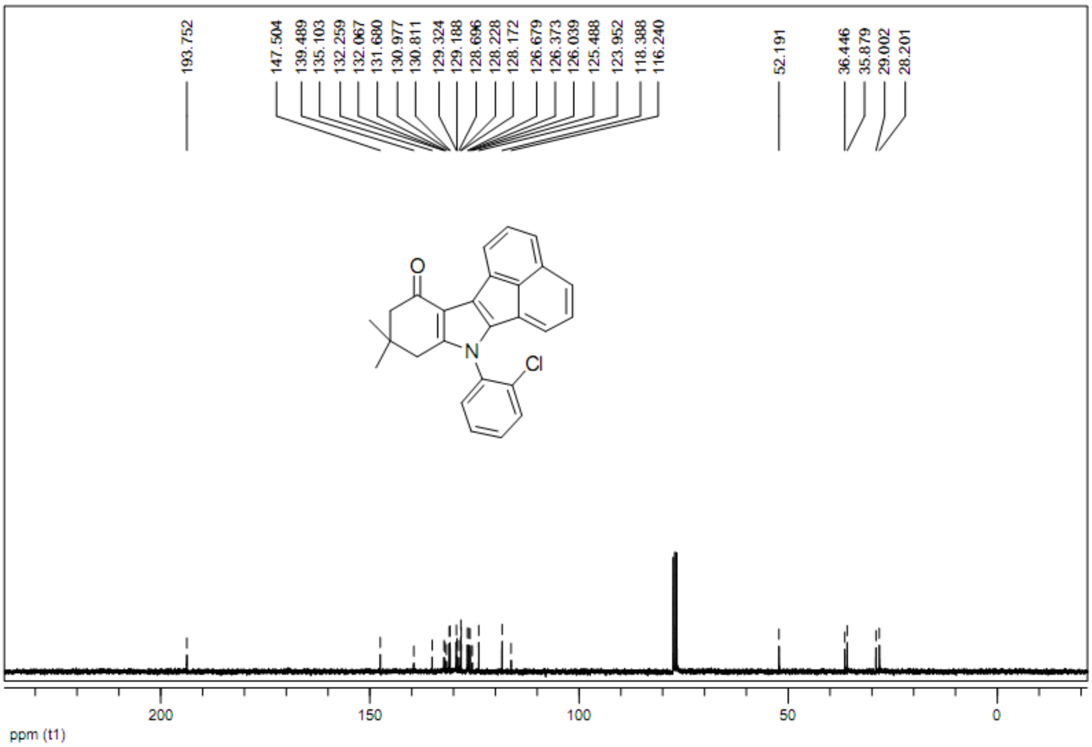

Figure S16. ^{13}C-NMR spectrum of compound **3h**

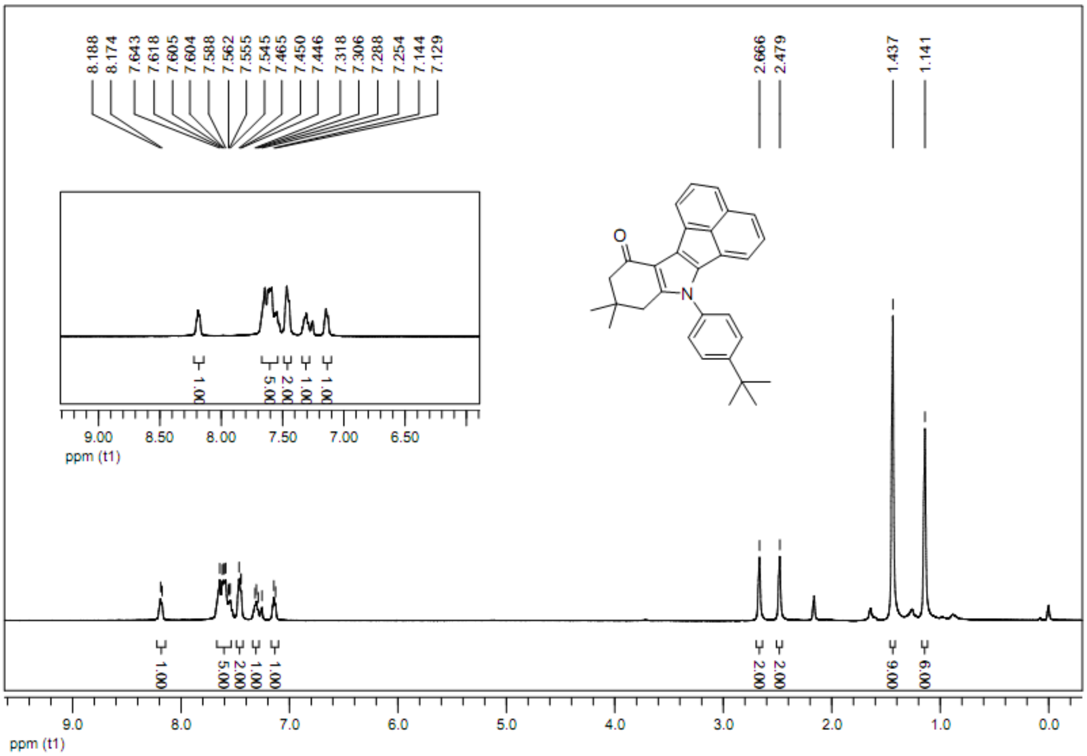

Figure S17. [13]H-NMR spectrum of compound **3i**

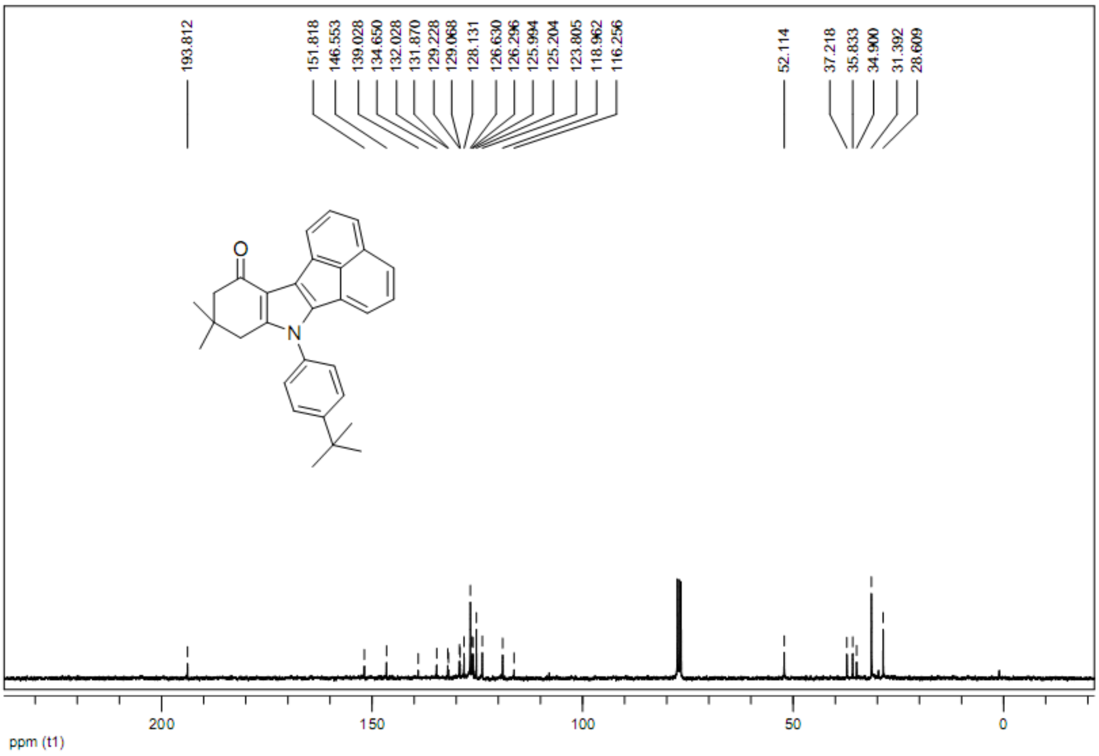

Figure S18. ^{13}C-NMR spectrum of compound **3i**

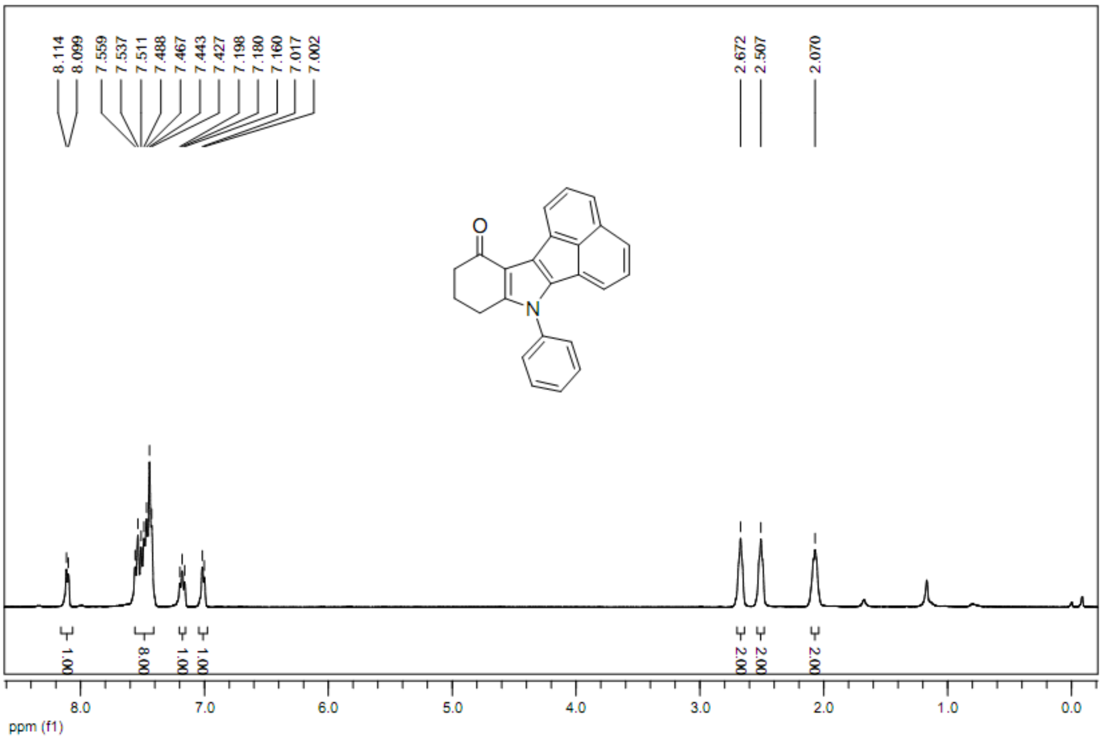

Figure S19. ^{1}H-NMR spectrum of compound **3j**

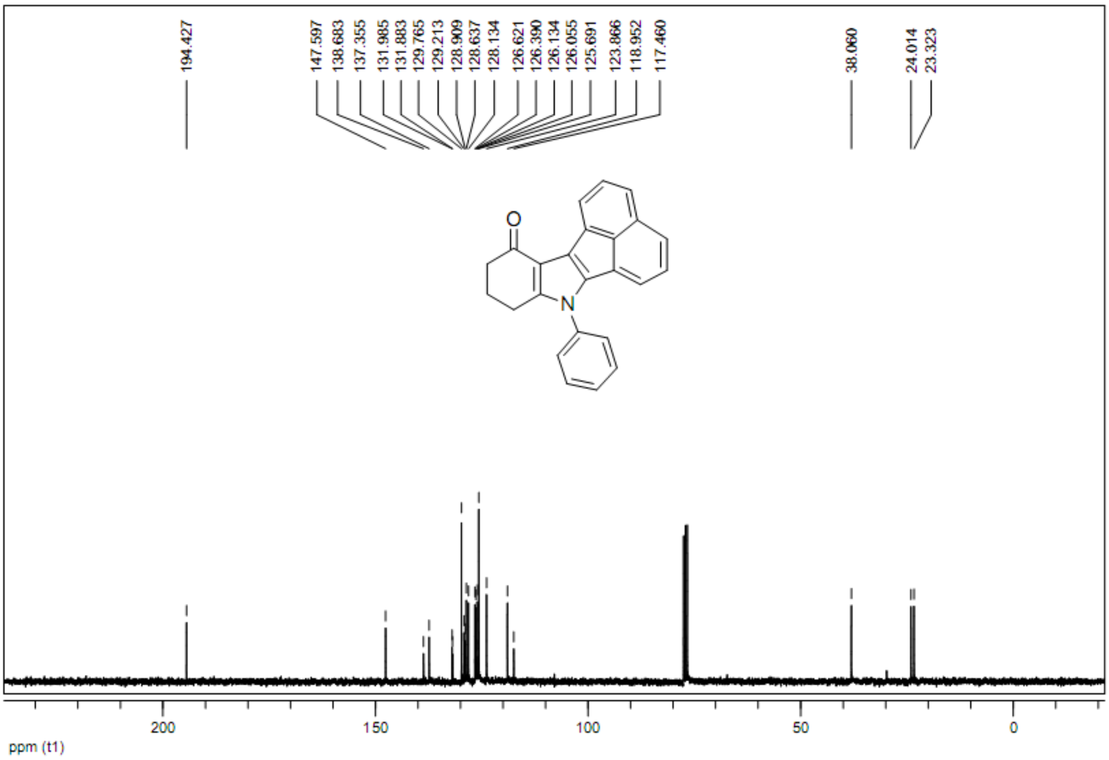

Figure S20. ^{13}C-NMR spectrum of compound **3j**

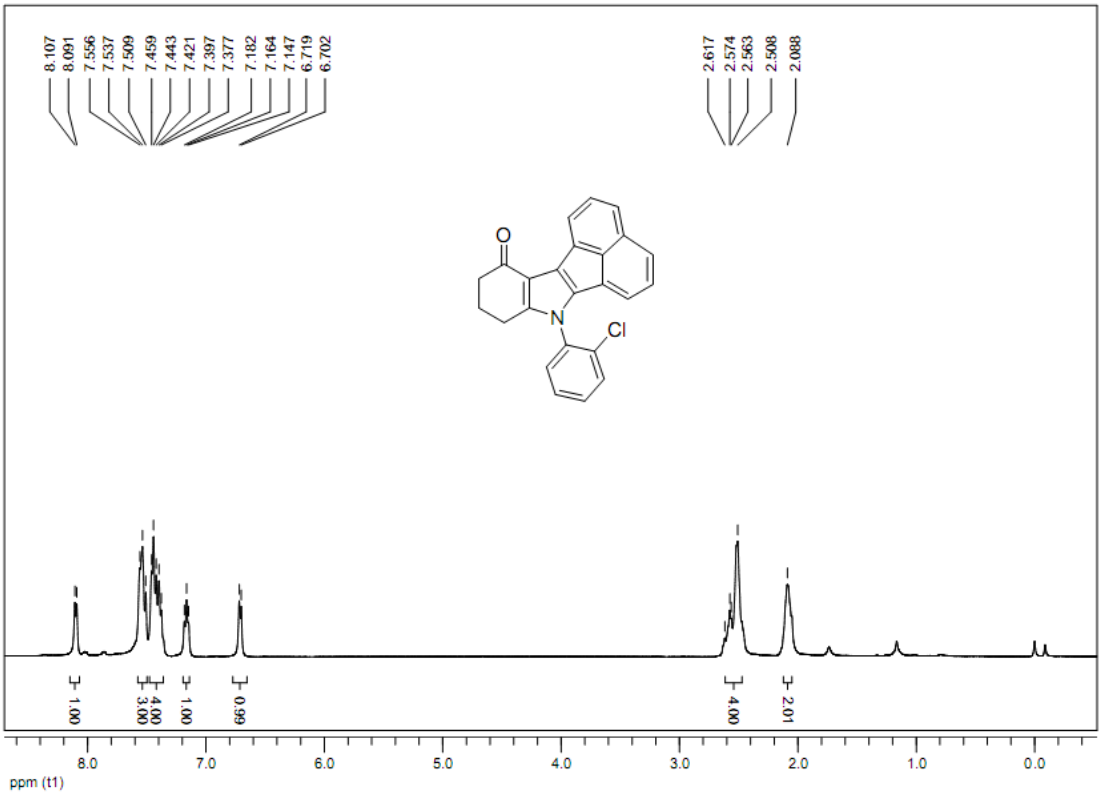

Figure S21. ^{1}H-NMR spectrum of compound **3k**

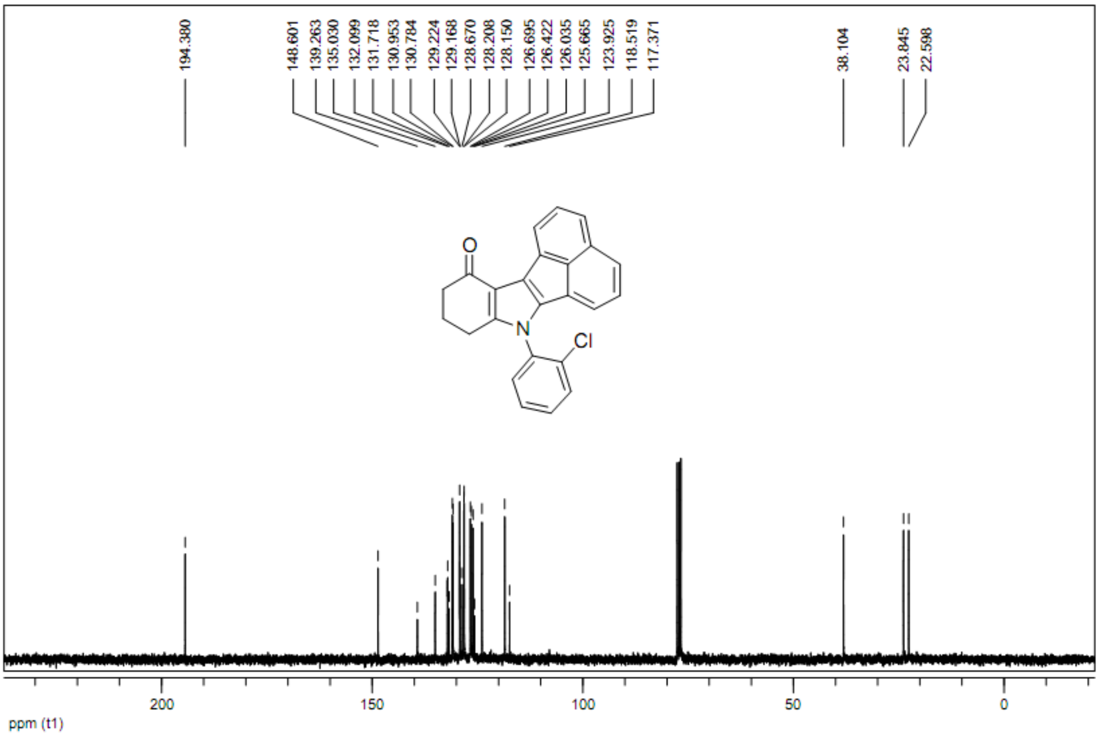

Figure S22. ^{13}C-NMR spectrum of compound **3k**

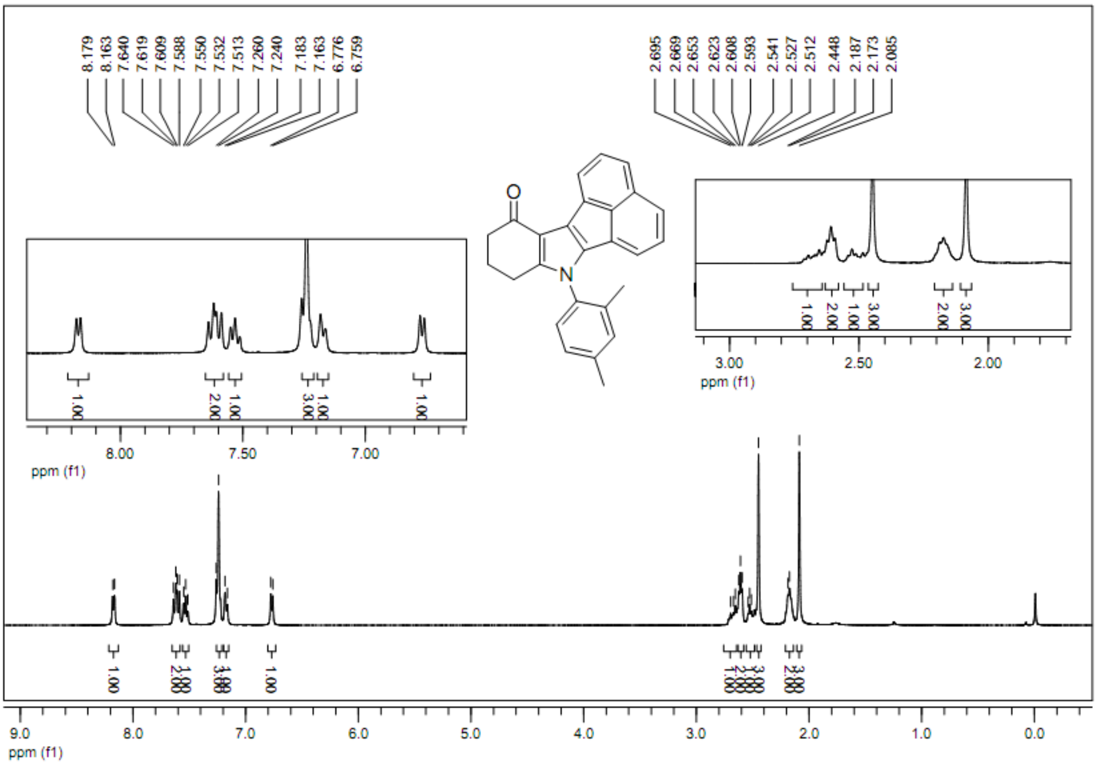

Figure S23. ^{1}H-NMR spectrum of compound **3l**

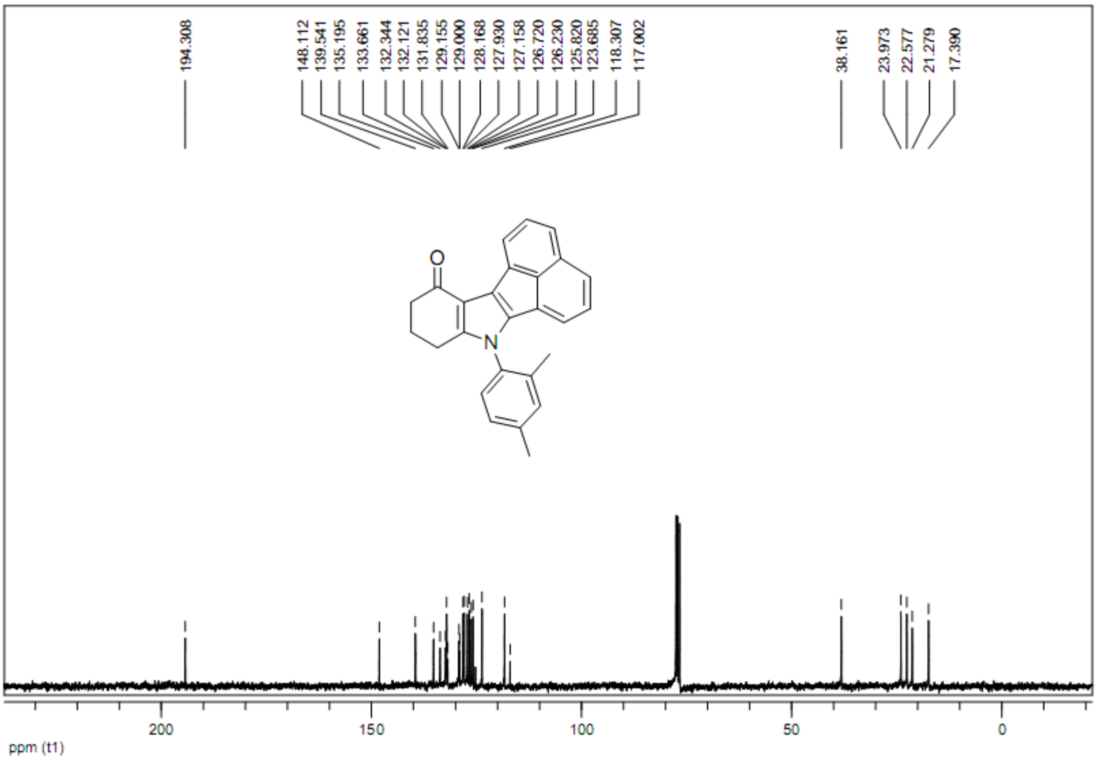

Figure S24. ^{13}C-NMR spectrum of compound **3l**

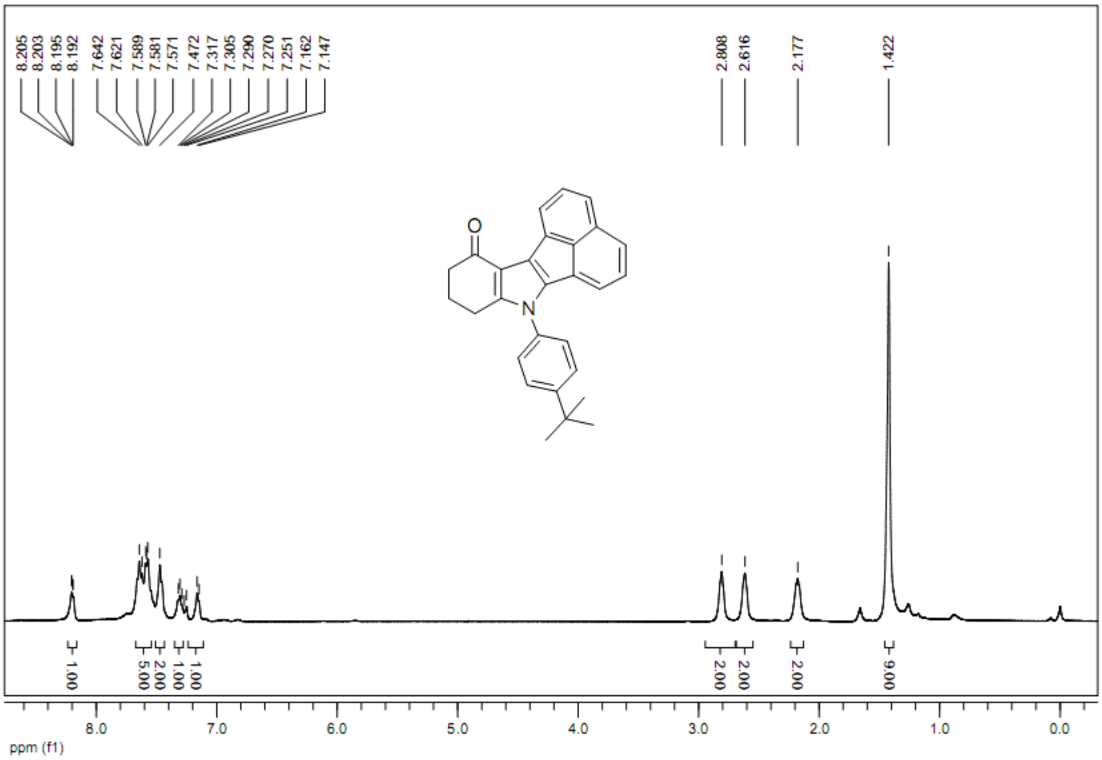

Figure S25. ^{13}C-NMR spectrum of compound **3m**

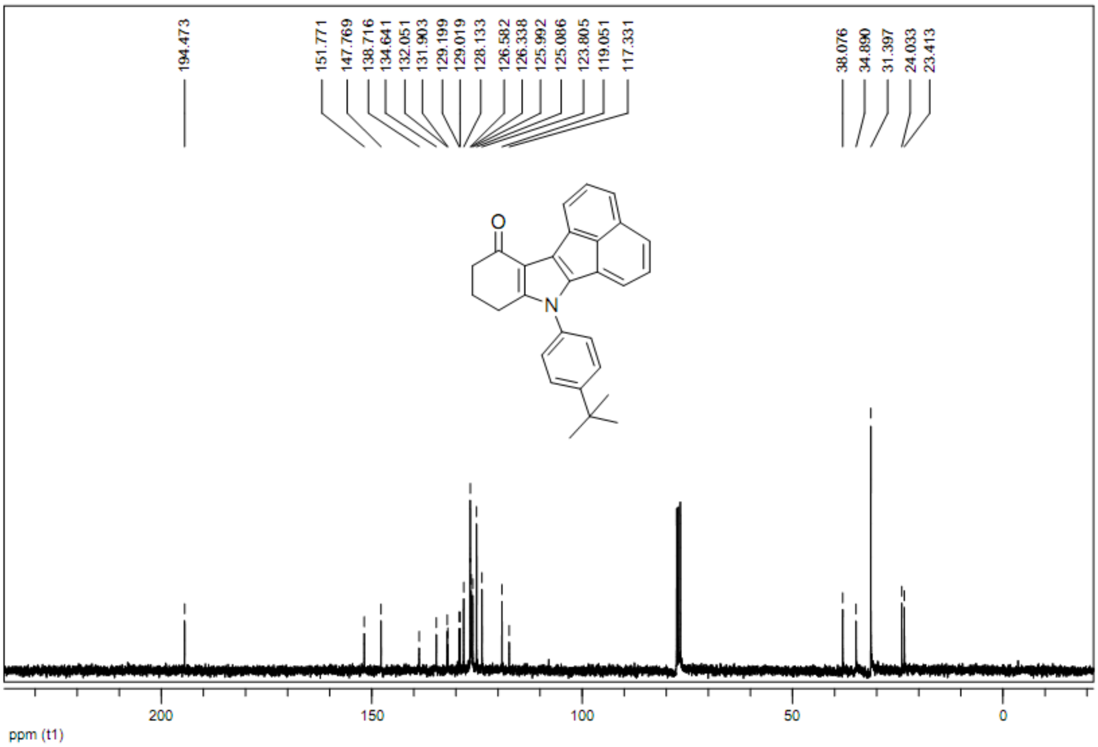

Figure S26. ^{13}C-NMR spectrum of compound **3m**

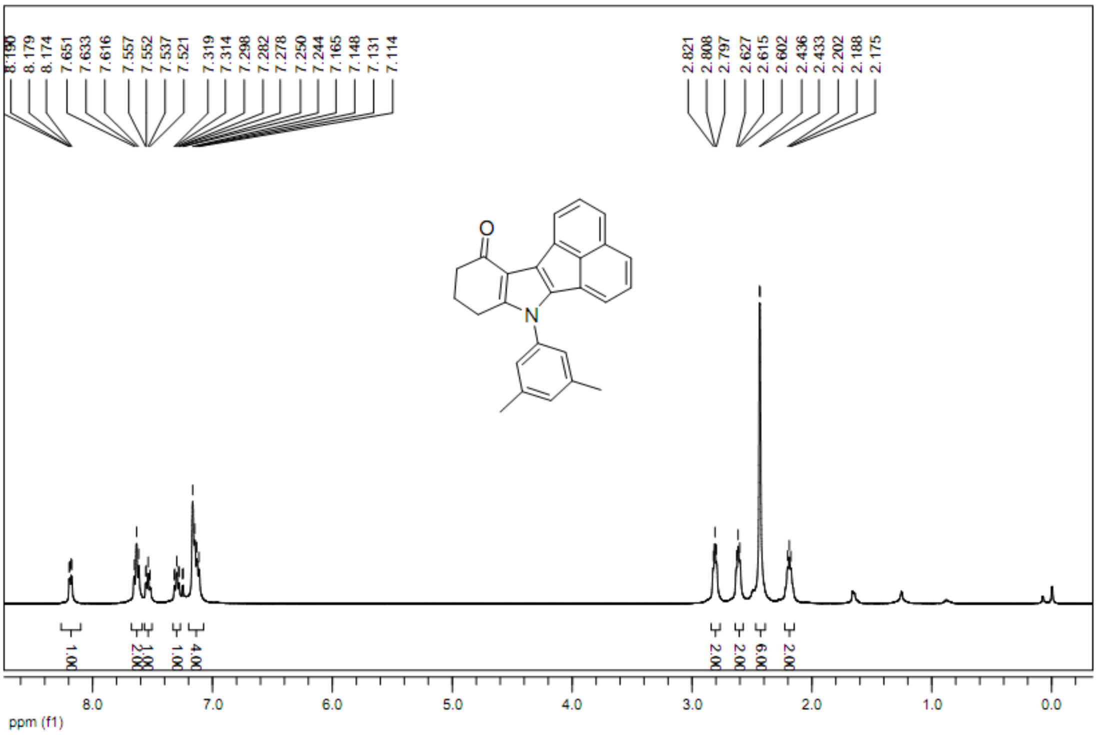

Figure S27. ^{1}H-NMR spectrum of compound **3n**

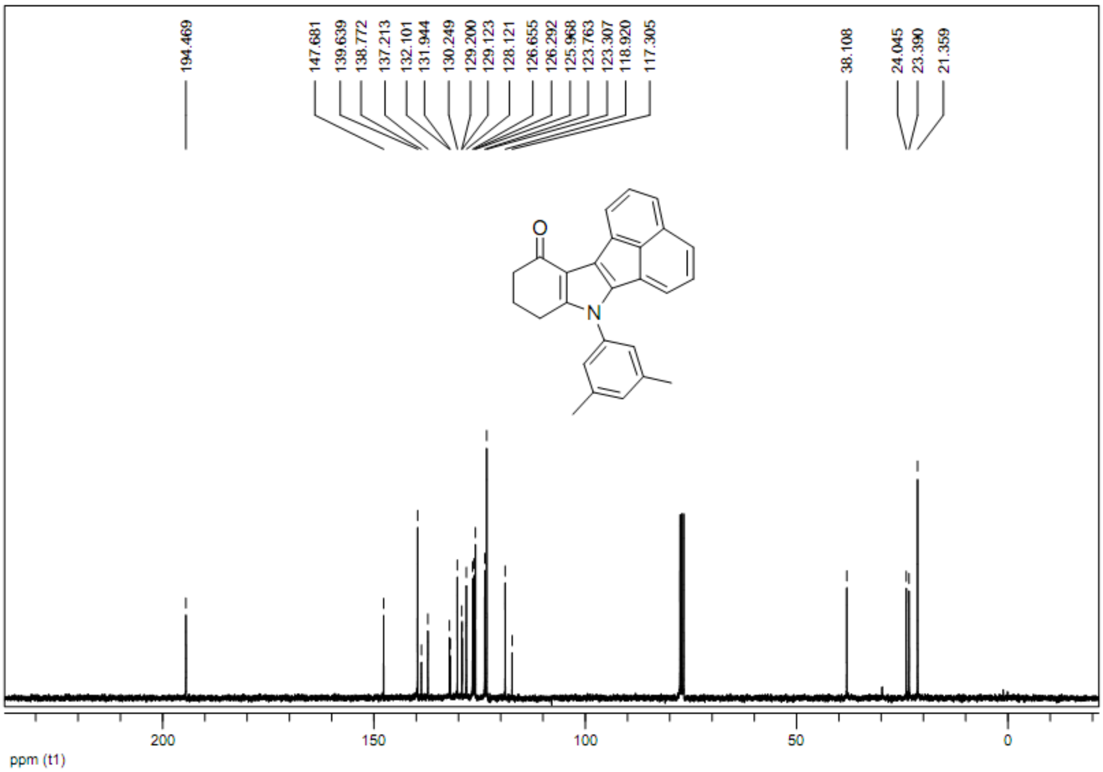

Figure S28. ^{13}C-NMR spectrum of compound **3n**
